# SumSec: Accurate Prediction of Sumoylation Sites Using Predicted Secondary Structure

**DOI:** 10.3390/molecules23123260

**Published:** 2018-12-10

**Authors:** Abdollah Dehzangi, Yosvany López, Ghazaleh Taherzadeh, Alok Sharma, Tatsuhiko Tsunoda

**Affiliations:** 1 Department of Computer Science, Morgan State University, Baltimore, MD 21251, USA; 2 Genesis Institute of Genetic Research, Genesis Healthcare Co., Tokyo 150-6015, Japan; 3 School of Information and Communication Technology, Griffith University, Gold Coast 4222, Australia; ghazaleh.taherzadeh@griffithuni.edu.au; 4 Institute for Integrated and Intelligent Systems, Griffith University, Brisbane 4111, Australia; alokanand.sharma@riken.jp; 5 School of Engineering & Physics, University of the South Pacific, Suva, Fiji; 6 Laboratory for Medical Science Mathematics, RIKEN Center for Integrative Medical Sciences, Yokohama, Kanagawa 230-0045, Japan; tatsuhiko.tsunoda@riken.jp; 7 CREST, JST, Tokyo 102-0076, Japan; 8 Department of Medical Science Mathematics, Medical Research Institute, Tokyo Medical and Dental University, Tokyo 113-8510, Japan

**Keywords:** post translational modification, sumoylation, ensemble classifier, bagging, secondary structure, profile-bigram

## Abstract

Post Translational Modification (PTM) is defined as the modification of amino acids along the protein sequences after the translation process. These modifications significantly impact on the functioning of proteins. Therefore, having a comprehensive understanding of the underlying mechanism of PTMs turns out to be critical in studying the biological roles of proteins. Among a wide range of PTMs, sumoylation is one of the most important modifications due to its known cellular functions which include transcriptional regulation, protein stability, and protein subcellular localization. Despite its importance, determining sumoylation sites via experimental methods is time-consuming and costly. This has led to a great demand for the development of fast computational methods able to accurately determine sumoylation sites in proteins. In this study, we present a new machine learning-based method for predicting sumoylation sites called SumSec. To do this, we employed the predicted secondary structure of amino acids to extract two types of structural features from neighboring amino acids along the protein sequence which has never been used for this task. As a result, our proposed method is able to enhance the sumoylation site prediction task, outperforming previously proposed methods in the literature. SumSec demonstrated high sensitivity (0.91), accuracy (0.94) and MCC (0.88). The prediction accuracy achieved in this study is 21% better than those reported in previous studies. The script and extracted features are publicly available at: https://github.com/YosvanyLopez/SumSec.

## 1. Introduction

Post Translational Modifications (PTMs) are enzymatic alterations of proteins after the translation process in which a macromolecule binds to a particular amino acid at a specific location [1]. These modifications play important roles in altering the functioning of proteins [2]. Proteins that undergo a PTM have been reportedly involved in a wide range of important biological interactions, including cell signaling, histone modification, subcellular localization, transcriptional regulation, apoptosis, protein stability, response to stress, in addition to mediating signal transduction, activating or deactivating enzymes and transporters, and underlying protein degradation and recycling [3,4,5,6,7].

The function of an altered protein is often dependent on the macromolecule by which it is bound. So far, the number of known PTMs is estimated to be over 200 [8]. In addition, the position of PTMs within the protein sequence is highly specific and depends on particular amino acids, motifs, and domains as well as the 3D structure of proteins [1]. Therefore, the identification of a PTM site (with respect to its particular type) can provide significant biological insights. Among different PTMs, sumoylation is one of the most recent and important types discovered thus far. It is defined as the binding of small ubiquitin-like modifier (or SUMO), which are small proteins in the cell. The most important types of such small proteins are SUMO1, SUMO2, and SUMO3 [9]. These proteins mainly bind to lysine, which is the most receptive amino acid to such macromolecules. Because of this, determining those lysines that constitute sumoylation sites is of particular interest. This PTM is particularly important due to its wide spectrum of critical cellular functions, such as nuclear-cytosolic transport, transcriptional regulation, protein stability, response to stress, neurodegeneration, immune-related diseases, and certain types of cancers [10,11,12,13,14,15,16,17]. 

Despite its aforementioned importance, the main method for accurately detecting PTMs and particularly sumoylation sites is experimental which turns out to be costly and time-consuming. Difficulties in isolating proteins, as well as diversity and molecular complexity of PTMs are two main obstacles in determining PTM sites using experimental method, effectively [18]. This issue becomes even more difficult for proteins with unknown structure. Considering the exponential growth in the number of sequenced proteins in the next generation sequencing era, there is a critical demand for fast and cost-effective computational methods able to accurately predict PTM sites.

To address this issue, new studies have been introduced to determine PTM sites using computational methods and in particular, machine learning techniques [19,20,21,22,23,24,25,26,27,28,29]. However, there have been a limited focus on predicting sumoylation sites prediction because of its novelty. To date, only a few sequence-based approaches have been proposed for predicting sumoylation sites. Early studies mainly focused on features directly extracted from the occurrence of amino acids and the use of simple classifiers for prediction purposes [30,31]. Xue et al. proposed SUMOsp (v1.0) which used a group-based prediction system (GPS) similarity clustering [30]. An updated version SUMOsp (v2.0) was later proposed. This version enhanced the GPS model in order to achieve a higher specificity and accuracy than the SUMOsp (v1.0) [32]. Next, SUMOhydro was built and proposed in [27] by using Support Vector Machine (SVM) classifier with original type of predictive feature (binary-encoded hydrophobicity pattern). Zhao et al. proposed a new approach called GPS-SUMO [33]. This algorithm presented an enhanced version of the GPS-based approach introduced in SUMOsp 1.0 and 2.0, now combined with a particle swarm optimization algorithm [33]. 

More recent studies have shifted their focus to employ more complex sets of features. For instance, Yavuz and Sazerman were the first that used predicted disorder and conformation flexibility for sumoylation site prediction [34]. Most recent studies employed the concept of pseudo amino acid composition (e.g., pSumo-CD and SUMO-LDA) for extracting evolutionary information. This concept revolutionized PTM research and achieved the best results in predicting sumoylation sites among the state-of-the-art studies [26,35].

Although there have been promising advances in this research field, the performance of current sumoylation site predictors remains very limited. One of the main reasons lies in the fact that the most effective features, which have been previously used to tackle similar problems, have yet to be explored for this specific task. One example of promising and effective characteristics, which happen to be overlooked, is structure-based features [36,37,38,39,40,41,42,43,44,45,46,47]. In addition, the amount of available experimental data has also been a contributory factor to the poor performance of early approaches. On top of this, the imbalance ratio between sumoylation and non-sumoylation sites makes these studies more biased towards determining non-sumoylation sites. The impact of unbalanced datasets can be clearly seen in previous studies, which have consistently reported high specificity and low sensitivity values. This high specificity is a clear indicator of the ability of the predictors to correctly detect non-sumoylation sites, while lacking the ability to accurately detect sumoylation sites as it is inferred from their low sensitivity values. 

In this paper, we propose a novel machine learning-based method called SumSec which aims at tackling the current challenges in sumoylation prediction tasks. To build the model, we extracted features based on the predicted secondary structure of the proteins. These features were calculated using a profile-bigram approach, and a sliding window to provide local structural information. To the best of our knowledge, the predicted secondary structure has never been used for predicting sumoylation sites. To avoid any bias towards non-sumoylation sites and increase the sensitivity of the predictor, the training data was first balanced. For prediction purposes, we employed an ensemble classifier based on the bagging technique. The prediction results demonstrate the effectiveness of SumSec and its unique ability to accurately predict sumoylation sites, thus outperforming current methods in the literature. SumSec demonstrates high sensitivity (0.91), accuracy (0.94), and Matthew’s correlation coefficient (0.88). The prediction accuracy achieved in this study is 21% higher than that reported for pSUMO-CD, the best sumoylation site predictor currently available.

## 2. Materials and Methods

### 2.1. Dataset Description

In this study we use the dataset that was constructed in [48]. This dataset consists of benchmarks related to 12 different types of lysine PTMs including sumoylation as a part of the Compendium of Protein Lysine Modifications (CPLM) database. This dataset has been recently updated and widely used in the literature [17,33,35]. This enables us to directly compare our results with previous studies found in the literature. The original sumoylation benchmark in the CPLM consists of 528 proteins with 928 sumoylation sites, in total. We first removed those sequences with >40% sequential similarity using CD-HIT [49]. The filtering of sequences with high sequential similarity guarantees the consistent performance of our model on remote homology samples. As a result of this filtering, 780 positive samples (sumoylated sites) and 21,353 negative samples (non-sumoylated sites) distributed across 448 proteins were retained. The difference between the number of positive and negative samples makes this benchmark quite unbalanced. This imbalance can strongly bias the performance of any predictor towards the identification of negative samples (a high true negative rate) over the detection of positive samples (a low true positive rate). This is the main reason why previously proposed models have consistently reported high specificity and low sensitivity values [27,30,35]. 

The two commonly used strategies to overcome the imbalance problem are over-sampling and under-sampling. The idea behind over-sampling is to duplicate the positive samples to increase them to the level of negative samples while in under-sampling, we delete some of the negative samples to decrease them to the level of positive samples. The over-sampling procedure could increase the probability of over-fitting the model due to multiplication of positive samples while under-sampling often provides a modest solution for a given model. Therefore, we selected under-sampling procedure to overcome the imbalance problem. As a result of under-sampling as it was done in [50], we ended up with 780 negative and 780 positive samples. In this way, we avoid bias in our benchmark towards negative samples and increase our chance to detect more positive samples or in other words, more sumoylation sites.

### 2.2. Predicted Secondary Structure

The functioning of proteins crucially depends on their tertiary structures which in turns depend on their secondary structures. The secondary structure of a protein is defined as how it folds locally to build local structure in terms of helical (α-helix), strands (β-strands), or unstructured connectors between these two (so called coil). The secondary structure of proteins can provide important information about the interaction of the amino acids along the protein sequence. It also indicates how they are exposed to other macromolecules depending on their position inside or on the surface of 3D structure of proteins [40,43,47,51,52,53]. Therefore, it is considered an important source for providing information regarding the possible interaction of amino acids along the proteins. Predicted secondary structure has been widely used for feature extraction to tackle different problems and demonstrated its effectiveness by attaining promising results [19,20,21,29,39,42,43,45,52,54,55,56,57].

In this study, we use the predicted secondary structure using SPIDER 2.0 to extract our features. SPIDER 2.0 was introduced in [52,58]. It is considered as one of the most accurate predictors of local structure of the proteins using deep learning architecture. As its outputs, it predicts secondary structure, Accessible Surface Area (ASA), Torsion Angles, Contact Number (CN) [59], and Half-Sphere Exposure (HSE) [60]. Its predicted structural properties have been widely used in previous studies [21,29,42]. In terms of secondary structure, SPIDER 2.0 produced a L × 3 matrix where *L* indicates the length of a protein sequence and the three columns are the respective likelihood contribution to each local structure which are demonstrated in terms of helix (*ph*), strand (*pe*) and coil (*pc*).

The correlation between protein secondary structure and PTM has been investigated in detail in [61]. They have showed that in fact there is a relation between PTM and different elements of secondary structure. According to [61], PTMs are more likely to occur at coil or unstructured region of proteins compared to α-helix or β-strand. Based on the finding reported in [61], we hypothesize that predicted secondary structure can be used as effective feature to predict PTM sites.

Here we also investigated the possible relation between sumoylation and protein secondary structure. To do this, we calculated the absolute Pearson correlation for the different secondary structure elements. The correlation between H and E for positive samples is |rp1| = 0.26, and the correlation between E and C is |rp2| = 0.17. When we computed the correlation for all the positive and negative samples, we found the correlation between H and E is |rc1| = 0.17, and E and C is |rc2| = 0.08. Therefore, secondary structures H and E correlated more for positive samples compared to the entire dataset (|rp1| > |rc1|). Similar results were obtained for E and C structural features; i.e., |rp2| > |rc2|. This shows that the secondary structure of sumoylation sites correlates and features derived encompassing structural properties play a role in lysine site detection.

### 2.3. Feature Extraction 

**Figure 1:**
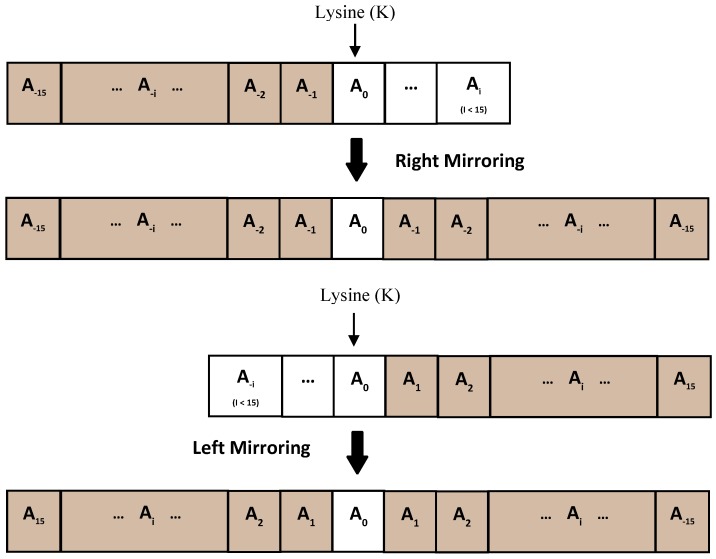
The method to extend the window for lysines in the terminus (under 14 amino acids upstream or downstream).

Here we extract two sets of features directly from the output of SPIDER 2.0 namely, predicted secondary structure occurrence (*SSpre-occur*) and profile-bigram (*SSpre-bigram*) from a neighboring window around each lysine (negative and positive samples). In this way, instead of a single amino acid, we extract more local information from its neighboring amino acids. In this study we use a window size of 31 amino acids (15 upstream and 15 downstream and the central lysine) as done in previous studies [19,20,21,22,62,63,64]. To apply windowing scheme for the lysines in terminus positions we adopted the mirror scheme to extend the window size [40,65]. Considering our adopted window size which is 15 upstream and downstream, the mirror effect will apply to those that are among the first and last 14 amino acids. If a lysine is positioned in terminus, the gap of 15 (upstream or downstream) amino acids is filled by the mirror effect of amino acids [65]. This method is shown in Figure 1.

We then extract our two sets of features namely *SSpre-occur* and *SSpre-bigram* for each lysine from its neighboring amino acids. The *SSpre-occur* feature set consists of the predicted secondary structure (*ph*, *pe*, *pc*) of each amino acid in the given neighboring window. In other words, for a given lysine, we extract the predicted secondary structure for all the 31 amino acid in its neighboring window. Therefore, we extract 93 features to build *SSpre-occur* (31 × 3 = *93*) feature group.

The *SSpre-bigram* feature set obtained using profile-bigram technique. Extracting profile-bigram from evolutionary or structural information was first introduced in [38,41]. This method was proposed to extract more information about local interaction of the amino acids and at the same time to avoid sparsity in extracted features compared to applying this method directly to protein sequence [41]. The *SSpre-bigram* is calculated in the following manner. Let SPIDER 2.0 matrix of size L × 3 be $S_{p}$. Each element $s_{pq}$ of matrix $S_{p}$ indicates the predicted secondary structure probabilities (*ph*, *pe*, and *pc*) for the *p^th^* amino acid. According to [41] matrix $S_{p}$ is represented by a profile bigram as:

(1) $$B_{p,q}=\overset{31}{\underset{k=1}{\sum }}s_{k,p}s_{k+1,q}$$

where 1≤p≤3 and 1≤q≤3.

**Figure 2:**
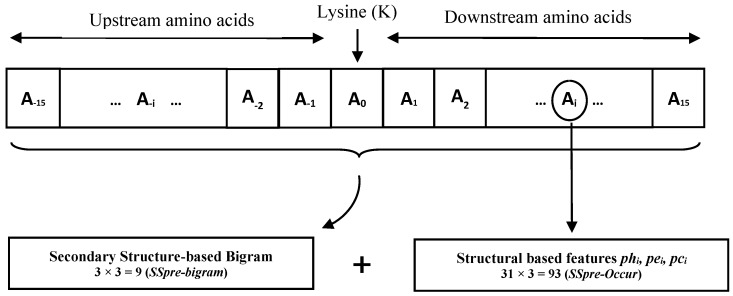
Our feature extraction scheme to build *SSpre-bigram* and *SSpre-Occur* feature sets.

This equation will return a 3 × 3 bigram frequencies $B_{p,q}$ (for p=1,2,3 and q=1,2,3). Thus, the bigram occurrence matrix B will consist of all the frequencies $B_{p,q}$. In this study, we have employed profile bigram because of its promising results [41,66,67,68,69,70,71,72,73]. Each bigram matrix B is then transformed to one feature vector as

(2) $$F={\left[B_{11},\ldots ,B_{ij},\ldots ,B_{3,3}\right]}^{T}$$

for i=1,2,3 and j=1,2,3, where superscript T denotes transpose. As a result, we build *SSpre-bigram* consisting of 9 features 3 × 3 matrix). The bigram feature extraction technique extracts an *n* × *n* matrix feature group regardless of the adopted window sizes. In other words, the number of features does not increase if the window size, increases. This is different from *SSpre-occur* in which its number of features directly related to the windows size. For our case, it extracts 9-dimensional feature vector in *SSpre-bigram* regardless of the window size adopted around a lysine residue. In this case, the bigram approach enables us to increase the window size around lysines without necessarily increasing the number of features. Our proposed feature extraction scheme is shown in Figure 2.

As a result, we extract 102 features in total (93 + 9) to build our feature vector using *SSpre-occur* and *SSpre-bigram* feature sets.

The datasets and scripts used in this study are publicly available online at: https://github.com/YosvanyLopez/SumSec.

### 2.4. Classification

**Figure 3:**
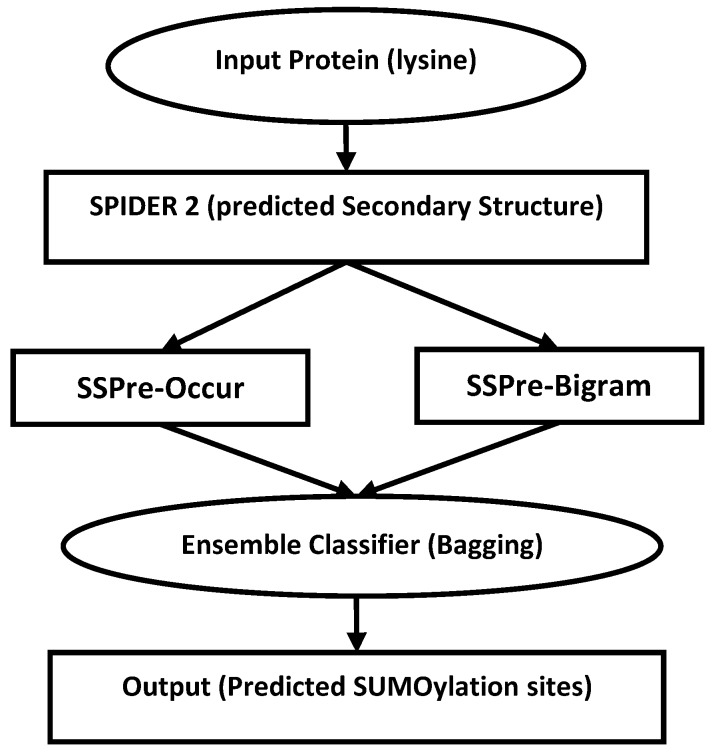
The general architecture of SumSec. The input protein is given to Spider 2.0 to predict its local structure. We then extract our features for each lysine residue and train a Bagging classifier to for sumoylation sites prediction.

In this study, we use straightforward and powerful ensemble classifier known as bagging to build our model. Bagging was first introduced in [74]. It is based on the idea of dividing the input data into certain number of subsample sets called bootstrap, applying a base learner to each bootstrap, and then aggregate those base classifiers using voting scheme. Such a model can increase diversity in the ensemble learner to enhance the prediction accuracy [75]. Here we use C4.5 decision tree as base learner [76]. C4.5 is the extension of ID3 decision tree based on the idea of adopting gain ratio instead of entropy to enhance the prediction performance [76,77]. Despite its simplicity, C4.5 has been shown as an effective classifier used with Bagging technique [74]. Here we tried different numbers of base learners for our model and among them, using 10 base learners attained the best results. Therefore, we use this number of base learners to build our ensemble model. To train the predictor, we used the Python implementation of decision trees. The general architecture of our model is shown in Figure 3.

### 2.5. Evaluation Method and Performance Measurements

To be able to directly compare our model with previous studies found in the literature, we adopted *k*-fold cross validation method. In k-fold cross validation, we first divide our data into *k* equal size subsets. We then train our model using the combination of *k-1* subsets and use the remaining one set to test our model. We repeat this process *k* times and until all the subsets are used for the test. In this way, we were able to use the available data in a more efficient way to validate our model [78]. To be able to evaluate our model for different cases, we conducted our *k*-fold cross validation using *k* equal to 6, 8, and 10.

We also adopted five most popular measurement criteria namely, sensitivity, specificity, accuracy, Matthew’s Correlation Coefficient (MCC), and Receiver-Operating Characteristic (ROC) curve [79]. These criteria are adopted here to provide more insights into the performance of our proposed model. The sensitivity, specificity, accuracy, and MCC are calculated as follows:

(3) $$Sensitivity=\frac{TP}{FN+TP}$$

(4) $$Specificity=\frac{TN}{FP+TN}$$

(5) $$Accuracy=\frac{TN+TP}{TN+FN+TP+FP}$$

(6) $$MCC=\frac{\left(TP\;\times \;TN\right)-\left(FP\;\times \;FN\right)}{\sqrt{\left(TN+FP\right)\;\times \;\left(TP+FN\right)\;\times \;\left(TP+FP\right)\;\times \;\left(TN+FN\right)}}$$

where TP is true positives, FP is false positives, FN is false negatives and TN is true negatives. The last evaluation metric used was the area under receiver-operating characteristic (ROC) curve. The curve is computed as the relation between sensitivity and False Positive Rate (FPR) changes at a range of different cut-offs. Here, the false positive rate is defined as follows:

(7) $$FPR=\frac{FP}{TN+FP}$$

Accordingly, the area under the ROC curve (AUC) is defined as follows:

(8) $$AUC\left(M\right)=\int_{\infty }^{-\infty }Sens\left(M\right)\times \left(-FPR{}^{\prime }\left(M\right)\right)dM$$

where *M* is the cut-offs of class prediction probability.

## 3. Results and Discussion

Our experimental results are shown in Table 1. To be able to directly compare our results with previous studies, we also present the prediction performance of pSumo-CD in this table. pSumo-CD is currently considered as the most accurate methods for sumoylation site prediction problem and outperforms all the other method found in the literature. The comparison was done by annotating all proteins in our dataset using the pSumo-CD web server. We report sensitivity, specificity, accuracy, and MCC metrics in Table 1 for all our experimentations as well as for pSumo-CD. As it is shown in this table, we achieve over 0.9 sensitivity, 0.96 specificity, 93% accuracy, and up to 0.88 for MCC using 10-fold cross validation. The results achieved using 6 and 8 folds cross validation are also consistent and very similar to the results achieved for 10-fold cross validation procedure. The small difference between performances achieved for different *k*-folds validation indicates the generality of our model and small possibility of overfitting.

As it is shown in Table 1, SumSec is able to successfully outperform pSumo-CD for all four evaluation criteria. The enhancement is significant and over 0.35 for sensitivity, over 0.2 for specificity, and 0.35 for MCC. We even achieve better specificity than pSumo-CD. It is considering that pSumo-CD does not balance the data and has a very high specificity compared to its sensitivity. It is the main reason why they reported very low MCC. However, our model successfully overcome this issue and achieves high sensitivity and specificity, simultaneously. Notably, for the first time we achieve over 93% prediction accuracy for sumoylation prediction site which is 21% better than those reported by pSumo-CD. These significant improvements for all the four measurement criteria demonstrate the preference of SumSec over pSumo-CD and its promising performance for sumoylation site prediction problem.

**Figure 4:**
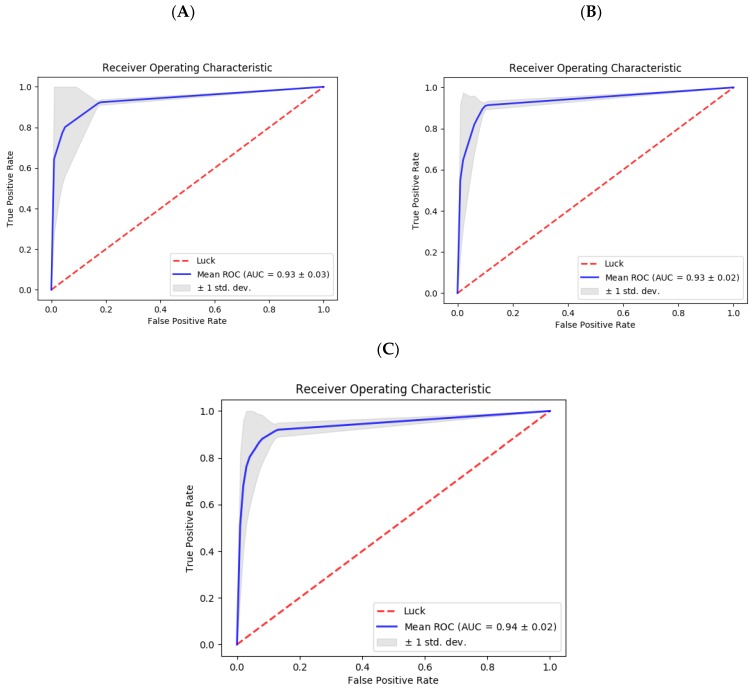
Receiver operating characteristic curves of SumSec performance. Three panels show results for 6-fold (**A**), 8-fold (**B**), and 10-fold (**C**) evaluation schemes.

To provide more insight into the performance of SumSec, we generated ROC curve to measure AUC (area under the curve). The results of the ROC-AUC analysis of our method for 6, 8, and 10-fold cross validations are shown in Figure 4. The average AUC value for all fold numbers was recorded at 0.93 (0.94 for 10-fold cross validation) indicating stable performance of SumSec. In all cases, the higher standard deviation was associated with lower score cut-offs.

Our achieved results demonstrate the effectiveness and accuracy of SumSec in predicting sumoylation sites. High sensitivity of our model compared to those that were reported in previous study demonstrates the capability of SumSec to specify those lysines that undergo sumoylation modification. Our results also demonstrate the effectiveness of structural-based features and in particular predicted secondary structure for our case to predict sumoylation sites.

Note that since sumoylation site prediction task is a new problem of interest, there is no other benchmark available to be used as an independent test set. Therefore, there is a limitation to fully investigate the generality of SumSec or any other predictor. Considering this limitation, we proceeded with the same method that was used in the previous studies to evaluate SumSec to be able to directly compare our results with the previous studies. However, to avoid overfitting, we conducted all our tuning on an independent validation set. On top of that, we avoided using feature extraction or use of complicated models with lots of parameters to tune to avoid reusing this benchmark. We also validated our model with more than one cross-validation scheme to make sure our results are consistent. To further investigate the generality of SumSec, in future we aim at validating our model on an independent test set. We will actively communicate with experts in the field to build a new sumoylaiton benchmark to be used as independent test set.

## 4. Conclusions and Future Work

In this study, we proposed a new machine learning model called SumSec to predict sumoylation sites as one of the most important post translational modifications. To do this, we first extracted structural features from the predicted secondary structure using the concept of profile-bigram. To the best of our knowledge, predicted secondary structure has never been used for this task. We then employed Bagging classifier to our extracted features to build the SumSec. All our experimentation was conducted on a benchmark with less than 40% sequential similarity to make sure our method is applicable for remote homology sequences. We also balanced our benchmark to avoid overfitting and bias towards predicting just negative samples. 

Our achieved results indicated that SumSec is capable of significantly outperforming all the previously proposed models for this task. We reported 0.91, 0.96, and 0.88 sensitivity, specificity, and MCC which are significantly better than those reported in the literature for sumoylation site prediction task. SumSec also achieved 93.8% prediction accuracy which is 21% better than reported result by pSumo-CD.

Considering the promising results using predicted secondary structure, we aim at investigating other aspects of structural information such as ASA, HSE, and torsion angle for feature extraction to achieve further enhancement for sumoylation site prediction problem. We also aim at employing these features to predict other PTM types.

## Figures and Tables

**Table 1:** The results achieved in this study for SumSec for 6, 8, and 10-fold cross validation.

Methods	Sensitivity	Specificity	Accuracy	MCC
*pSumo*-CD	0.536	0.921	72.8%	0.494
C-Validation 6	**0.910**	0.959	93.5%	0.873
C-Validation 8	0.907	0.963	93.4%	0.872
C-Validation 10	**0.910**	**0.967**	**93.8%**	**0.880**

Compared to pSumo-CD. Note that pSumo-CD results are also evaluated using 10-fold cross validation.

## Abbreviations

PTM	post-translation modification
SumSec	Sumoylation predictor using Structural-based features
ASA	accessible surface area
FN	false negative
TN	true negative
TP	true positive
FP	false positive
MCC	Matthew’s correlation coefficient
AUC	area under curve
ROC	receiver operating characteristic
